# Case report: Clinical features of pediatric acute myeloid leukemia presenting with cardiac tamponade: a case series study and literature review

**DOI:** 10.3389/fonc.2024.1391768

**Published:** 2024-06-13

**Authors:** Tonghui Li, Xue Tang, Xuezhi He, Lei Zhang, Ya Zhang, Lulu Wang, Shilin Liu, Guichi Zhou, Feiqiu Wen, Sixi Liu, Huirong Mai, Ying Wang

**Affiliations:** ^1^ Department of Hematology and Oncology, Shenzhen Children’s Hospital, Shenzhen, Guangdong, China; ^2^ Department of Hematology and Oncology, Shenzhen Children’s Hospital of China Medical University, Shenzhen, Guangdong, China; ^3^ Ultrasound Diagnosis Department, Shenzhen Children’s Hospital, Shenzhen, Guangdong, China; ^4^ Department of Pediatric Intensive Care Unit, Shenzhen Children’s Hospital, Shenzhen, Guangdong, China; ^5^ Radiology Department, Shenzhen Children’s Hospital, Shenzhen, Guangdong, China

**Keywords:** pediatric, acute myeloid leukemia, cardiac tamponade, cardiac myeloid sarcoma, pericardial effusion

## Abstract

**Objective:**

This study aims to elucidate the clinical features observed in cases of pediatric acute myeloid leukemia (AML) initially presenting with cardiac tamponade and to share treatment experiences.

**Materials and methods:**

Five pediatric patients were initially diagnosed with AML accompanied by cardiac myeloid sarcoma (MS). The diagnosis was established by examining our hospital records and reviewing pertinent literature from 1990 to July 2023, accessible through MEDLINE/PubMed. We comprehensively assessed the clinical characteristics and treatment modalities employed for these patients.

**Result:**

Five pediatric patients presented with acute symptoms, including shortness of breath, malaise, cough, and fever, leading to their hospitalization. Physical examination revealed irritability, hypoxia, tachypnea, tachycardia, and hypotension. Initial detection utilized chest X-ray or echocardiogram, leading to subsequent diagnoses based on pericardial effusion and/or bone marrow examination. Two patients received chemotherapy at the time of initial diagnosis, one with cytarabine and etoposide, and the other with cytarabine and cladribine. Post-treatment, their bone marrow achieved remission, and over a 2.5-year follow-up, their cardiac function remained favorable. Unfortunately, the remaining three patients succumbed within two weeks after diagnosis, either due to receiving alternative drugs or without undergoing chemotherapy.

**Conclusion:**

This is the first and largest case series of pediatric AML patients with cardiac MS, manifesting initially with cardiac tamponade. It highlights the rarity and high mortality associated with this condition. The critical factors for reducing mortality include identifying clinical manifestations, conducting thorough physical examinations, performing echocardiography promptly, initiating early and timely pericardial drainage, and avoiding cardiotoxic chemotherapy medications.

## 1 Introduction

Cardiac tamponade is a life-threatening condition due to its inherent risk of complications that can lead to sudden cardiac arrest. It is typically induced by pericardial effusions or cardiac tumors and can manifest in a variety of cardiac and non-cardiac conditions (1). Pericardial effusion occurs in less than 0.5% of patients with acute myeloid leukemia (AML) (2). AML constitutes 15% to 20% of pediatric acute leukemias, with extramedullary manifestations known as myeloid sarcoma (MS). However, extramedullary sarcomas involving the heart are extremely rare in children (3). Given the limited number of reported cases in the literature, there are currently no definitive diagnostic and therapeutic guidelines for AML presenting with cardiac tamponade. Worldwide, hematologists rely on their clinical experience to identify and manage such cases.

This study aims to report a case of pediatric AML with cardiac tamponade as the initial manifestation and retrospectively analyze all reported cases of AML with cardiac tamponade as the initial presentation in recent years. The objective is to explore these cases’ clinical characteristics, treatment approaches, and prognosis.

## 2 Patients and methods

Clinical data were collected from our hospital, and a literature review was conducted. We reviewed all the publications using the Chinese Wan Fang Database, the China National Knowledge Infrastructure, the China Science and Technology Journal Database, PubMed, and Google Scholar for citations published from January 1990 to July 2023. The search terms were pediatric, acute myeloid leukemia, cardiac tamponade, cardiac MS and pericardial effusion. We collected 5 pediatric AML who first presented with cardiac tamponade for our analysis (4–7), including the patient from our hospital (case 1).

## 3 Results

### 3.1 Clinical features of pediatric AML patients with initial manifestation of cardiac tamponade

**Table 1** summarizes the characteristics of all five patients diagnosed with AML who initially presented with cardiac tamponade. This summary is based on our study and includes a review of cases reported between January 1990 and July 2023.

**Table 1:** Clinical features of AML pediatric patients with initial presentation of cardiac tamponade.

Case No.	Age/Sex	Major symptoms and signs	FAB Subtype	Cytogenetics/Molecular Marker	Heart involved	Diagnostic modality	Therapy	Outcome
1	2/F	Decreased appetite, irritable; respiratory distress, tachycardic, and hypotensive;	M5	47, XX, +8, t (9; 11)(p21; q23); *KMT2A::MLLT3*(+), ASXL1(+), FLT3-TKD(+);	Left ventricular apex and left atrium	Echocardiogram; Pericardial fluid; BMA;	Induction: cytarabine, etoposide, cladribine and rhG-CSF; Maintenance: Venclexta and Gilteritinib	CR Alive
2 (4)	7/M	Respiratory distress;	M5	Not mentioned	Myocardium, sinoatrial and atrioventricular nodes	CXR; Echocardiogram; BMA;	No chemotherapy	Died of cardiac arrest
3 (5)	1.4/F	Barking cough, progressive shortness of breathing,sudden onset of lip cyanosis; Facial swelling and tachypnea;	M4	51,XX,add(1)(p32),del(4) (q31),+del(6)(q23),add(7) (q11.2),+8,+8,der(11) t(7;11)(q11.2;q23),+13, add(16)(q24), +19; FISH: *MLL-r*	Pericardial cavity	CXR; Pericardial fluid; Cervical lymph node biopsy; BMA	Induction:vincristine,epirubicin, cytarabine and idarubicin; **Consolidation:** cytarabine, idarubicin, and etoposide;	Died from sepsis
4 (6)	2/F	Irritable, respiratory distress; Mild periorbital edema and edema of the hands and feet;	M5	Not mentioned	Myocardium and the apex of the right ventricle	CXR; Echocardiogram; Pericardial fluid; BMA	Systemic chemotherapy and radiation (drugs not mentioned)	Died from sepsis
5 (7)	5/M	Pallor, anasarca, tachypnea; Elevated jugular venous pressure, hepatomegaly, and a short mid-diastolic murmur at the tricuspid area;	M4	inv(16)	Mass in right atrium	CXR; Echocardiogram; BMA;	Induction**:**cytarabine and etoposide; **Consolidation**: 2 cycles of high-dose cytosine arabinoside with daunorubici;	CR Alive

CR, complete remission; CXR, Chest X Ray; ECMO, emergency extracorporeal membrane oxygenation; BMA, bone marrow aspiration.

The male-to-female ratio in this study was 2:3, and the age of diagnosis was within the range of 7 years old. Patients had no significant past medical history. In the case of our patient (case 1), a previously healthy and well-nourished 2-year-old girl, admission was prompted by a 4-day history of decreased appetite and malaise. The patient had an unremarkable medical and family history. She presented as irritable and hypoxic. On physical examination, she exhibited tachypnea, tachycardia, hypotension, hepatomegaly, jugular venous distension, and pulsus paradoxus. Additionally, an enlarged liver and palpable spleen tip were noted.

### 3.2 Auxiliary investigations in pediatric AML patients with initial manifestation of cardiac tamponade

In two cases (case 4 and case 5), hemogram analyses revealed anemia and leukocytosis. The biochemical findings indicated elevated lactate dehydrogenase levels. In our patient (case 1), the complete blood count displayed hyperleukocytosis with a white blood cell (WBC) count of 17.11×10^9^/L, hemoglobin (HB) of 11.7g/dL, and a platelet (PLT) count of 326×10^9^/L. The peripheral blood smear revealed 20% blasts. Biochemistry analysis showed elevated uric acid levels at 527.8 umol/L (normal range 90 to 310 U/L), lactate dehydrogenase at 1816 IU/L, and potassium at 2.69 mmol/L. Liver and renal functions, coagulation screen tests, and blood sugar were all within normal limits. Additionally, tests for HBsAg, anti-HCV Ab, anti-HIV Ab yielded negative.

**Figure 1:**
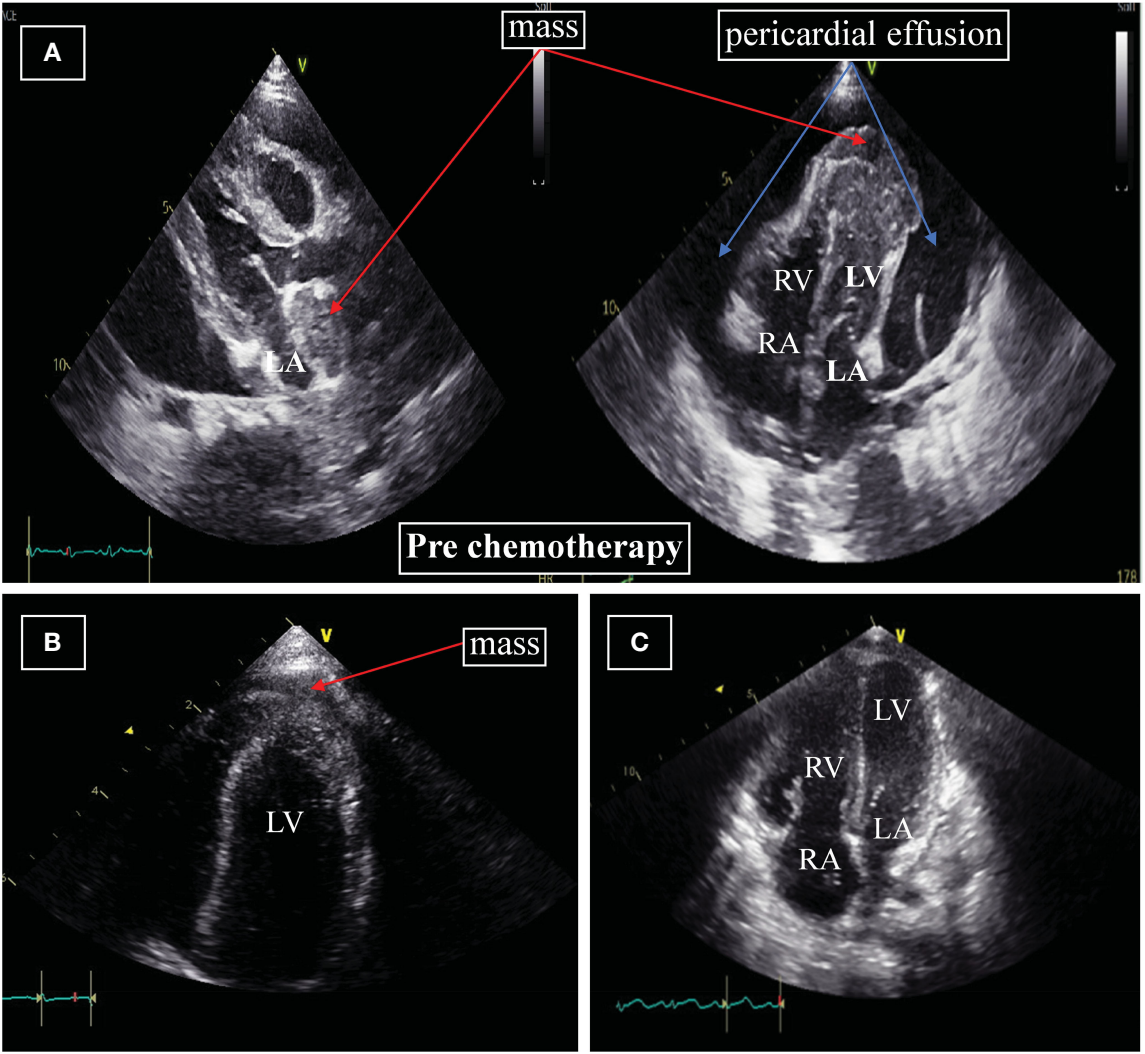
**(A)** Echocardiography at diagnosis. The apex of the left ventricle and left atrium displayed mass echoes protruding into the pericardial cavity, measuring 3.7×3.4cm and 2.3×1.2cm, respectively. **(B)** Echocardiography after four weeks of initiation of systemic chemotherapy. Significant reduction in the size of the mass to 2.2×0.7cm; the mass on the apex of the left atrium and pericardial effusion are no longer displayed. **(C)** Echocardiography after two months. The masses around the left ventricle and left atrium, pericardial effusion are not displayed. LA=left atrium; RA=right atrium; LV=left ventricle; RV=right ventricle.

**Figure 2:**
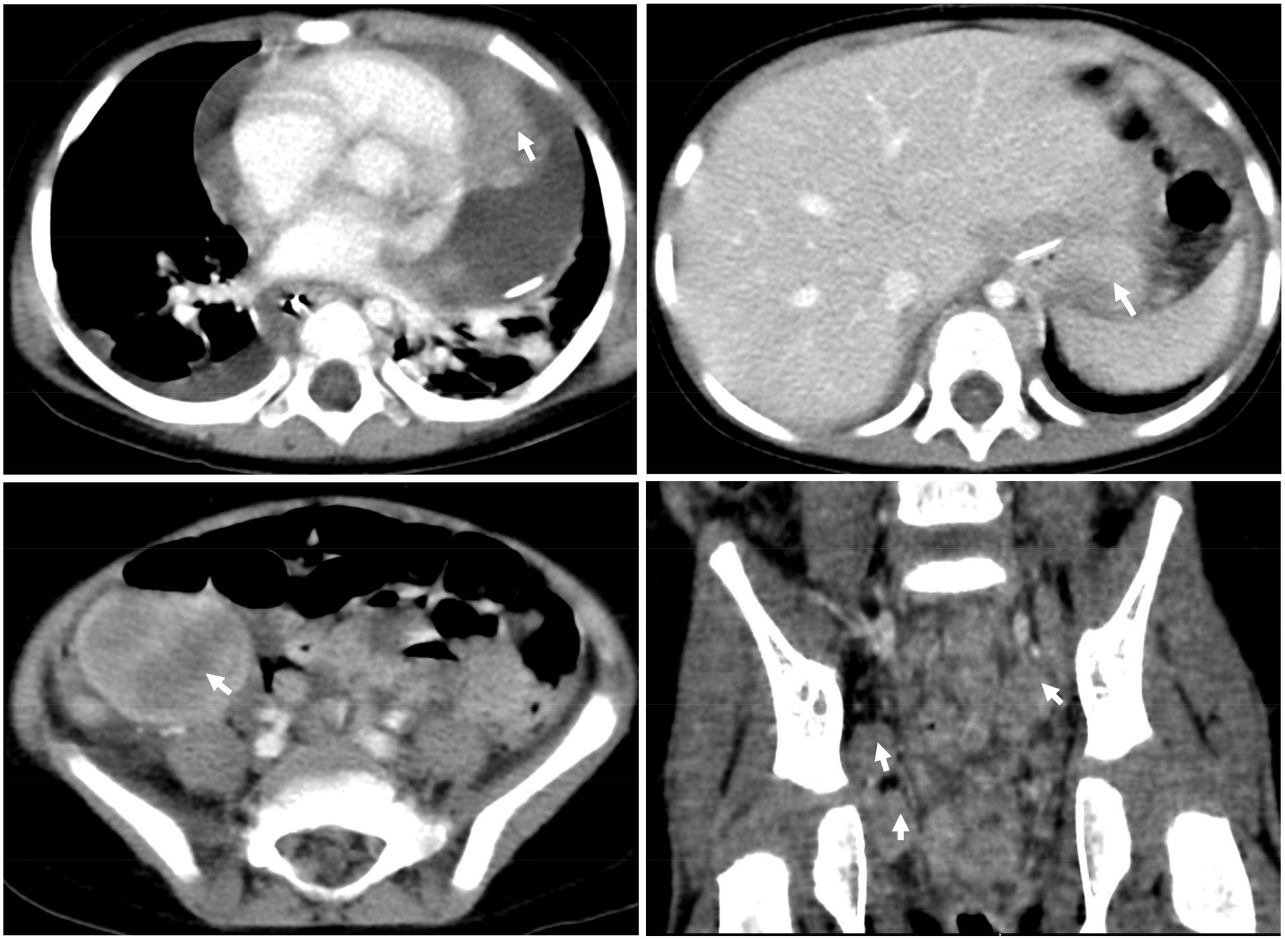
CT scan of the chest and abdomen at diagnosis. CT showed that not only the heart but also the chest, abdomen, and pelvis were involved.

Chest X-rays demonstrated a pronounced global enlargement of the cardiac silhouette in all cases. Alternatively, patients underwent echocardiography confirming the presence of a substantial pericardial effusion, cardiac occupation, and indications of tamponade. In our patient (case 1), the echocardiogram confirmed the presence of a large pericardial effusion and indicated limited diastolic and reduced systolic function. It indicates the presence of pericardial tamponade. The myocardium exhibited hypertrophy and inhomogeneity, characterized by multiple echogenic areas. Notably, the left ventricular apex and left atrium displayed mass echoes protruding into the pericardial cavity, measuring 3.7×3.4cm and 2.3×1.2cm, respectively (**Figure 1A**). The cardiac function was reduced, with an ejection fraction (EF) of 58%. CT showed that not only the heart but also the chest, abdomen, and pelvis were involved (**Figure 2**).

Subsequently, pericardiocentesis was performed on case 1, revealing leukemic blasts on fluid analysis. The pericardial effusion was bloody, containing 33.7×10^9^/L white blood cells, predominantly comprising mononuclear cells. Flow cytometry analysis of the pericardial effusion showed 11.9% abnormal myeloid cells expressing myeloperoxidase (MPO), CD33, CD13, CD56, CD4, CD38, CD64, CD65, and CD15, while lacking expression of CD7 and CD117. Additionally, bone marrow cytology revealed 46% immature mononuclear cells. Flow cytometry analysis of bone marrow cells indicated that these immature mononuclear cells expressed antigens consistent with those expressed by tumor cells in the pericardial effusion. Lumbar puncture confirmed the presence of central nervous system (CNS) leukemia. Chromosomal analysis of the bone marrow revealed the karyotype 47, XX, +8, t (9;11) (p21; q23) (8). Additionally, RNA sequencing (RNA-seq) identified the presence of the *KMT2A::MLLT3* fusion gene, along with ASXL1 and FLT3-TKD mutations at the molecular level.

All patients exhibited bone marrow involvement, with morphological and flow cytometry assessments indicating a classification of M4 or M5. Cytogenetic analyses, as presented in **Table 1**, did not reveal standard features among the cases.

### 3.3 Treatment and prognosis for pediatric AML patients with initial manifestation of cardiac tamponade

As shown in **Table 1**, pediatric patients with AML presenting with cardiac tamponade as the initial symptom are notably scarce. The associated mortality rate is high, and there is a lack of uniformity in the treatment approaches. In case of patient 5, chemotherapy was initiated with cytarabine and etoposide. Daunorubicin administration was deferred during the first cycle of induction due to the presence of mild left ventricular systolic dysfunction. Following the first cycle of induction chemotherapy, the solid mass diminished in size, and the obstruction was resolved, achieving bone marrow remission. Subsequently, the patient underwent consolidation chemotherapy with two additional cycles of high-dose cytosine arabinoside. The patient’s bone marrow sustained remission at the end of treatment and has exhibited favorable performance throughout the 2.5-year follow-up period, characterized by good cardiac function and the absence of any lingering sequelae.

Our patient (case 1) underwent pericardiocentesis, resulting in the gradual resolution of her dyspnea, tachycardia, and hypotension. After diagnosis, the hematologist initiated an individualized chemotherapy regimen based on the United Kingdom Medical Research Council protocol (7). This regimen comprised cytarabine (100 mg/m^2^ daily on days 1–6), etoposide (150 mg/m^2^ daily on days 3–5), cladribine (5 mg/m^2^ daily on days 6–9), and recombinant human granulocyte colony-stimulating factor (rhG-CSF) (5 ug/kg daily on days 9–19). Prolonged pericardial drainage significantly reduced the recurrence rate of cardiac tamponade (12% in patients with extended drainage vs. 52% in patients without extended drainage) (9). Though drainage volume gradually decreased after chemotherapy, the pericardial drainage was not removed until pericardial effusion was significantly reduced according to echocardiography. Additionally, on the third day of induction chemotherapy, the patient underwent lumbar puncture and intrathecal injection of chemotherapy drugs, including methotrexate, cytarabine, and dexamethasone. Three lumbar punctures were performed, and the second examination of cerebrospinal fluid revealed normal cells.

A follow-up echocardiogram conducted four weeks after initiating systemic chemotherapy demonstrated a significant reduction in the left ventricular apex mass (2.2×0.7cm) (**Figure 1B**), along with the normalization of other myocardial echogenicity and contractility. Two months later, the cardiac mass completely disappeared (**Figure 1C**), and bone marrow aspiration performed on day 50 of treatment did not show any residual leukemia. Moreover, MLL-AF9, ASXL1, and FLT3-TKD were negative. Her parents refused the second course of intravenous chemotherapy and hemopoietic stem cell transplantation (HSCT), considering her body’s inability to withstand it.

Considering RNA-seq revealed FLT3 mutation, she commenced treatment with Venetoclax and Gilteritinib to maintain treatment. She has been consistently monitored for over 2 years, with bone marrow remaining in remission and exhibiting good cardiac function.

## 4 Discussion

Pericardial effusions or cardiac tamponade is a relatively rare initial presentation in patients with hematologic malignancies. Lam KY et al.’s comprehensive review, covering over 12,000 consecutive autopsies conducted over a 20-year period, showed only a 4% incidence of cardiac involvement by leukemia (9). The majority of these cases remain undiagnosed in living individuals and are typically discovered only postmortem. This study reports the most significant series of pediatric AML patients who initially manifested with cardiac tamponade between 1990 and 2023.

Leukemic lung infiltration is more commonly observed in relapsed and refractory cases of AML (10). Since leukemic lung infiltrates typically exhibit an interstitial pattern on high resolution CT (HRCT) scans, most patients do not show clinical signs of respiratory distress despite the presence of lung infiltration upon admission (11). However, our patient presented irritable, hypoxic symptoms and signs of tachypnea, tachycardia, hypotension, hepatomegaly, jugular venous distension, and pulsus paradoxus. She was suspected of cardiac tamponade, combine with the diagnostic criteria for the cardiac tamponade of clinical diagnosis recommended by the European Society of Cardiology (12). Echocardiography can confirm a large pericardial effusion with signs of pericardial tamponade and prepare for pericardiocentesis. It is the first diagnostic method of choice in suspected cardiac tamponade and should be carried out without delay. Therefore, we believe the key is integrating clinical manifestations with a thorough physical examination to identify the issue and conduct echocardiography immediately. Following the scoring index introduced by Halpern et al. (13), a total score ≥6 warrants immediate pericardiocentesis in the absence of contraindications, as it can be life-saving. Our patient had a total score of 8, without severe coagulation disorders. Therefore, she underwent pericardial puncture and drainage immediately and was subsequently diagnosed with AML.

Our findings indicate that 80% (4 out of 5) of patients were diagnosed with pericardial effusion. MS may manifest in conjunction with either acute or chronic myeloid leukemia, with or without concurrent marrow disease at the time of diagnosis. Alternatively, it may occur independently as an isolated, non-leukemic, *de novo* condition or be associated with myeloproliferative neoplasms (14). Histopathology remains the gold standard for diagnosis, while image-guided techniques increase the biopsy yield. Guidelines and literature indicate that the pathology of body fluids can also be used as one of the diagnostic methods (15, 16). Therefore, examination of pericardial effusion and bone marrow puncture assumes a pivotal role in advancing the diagnosis process. When the child is in a life-threatening state, fluid examination via pericardiocentesis is safer than biopsy for promptly diagnosing the disease. Simultaneously, a comprehensive histopathological examination, including light microscopy, immunohistochemistry, and Fluorescence in Situ Hybridization (FISH), is imperative for biopsy samples.

The development of pericardial effusion often arises from the infiltration of malignant cells. Several factors associated with an increased incidence of extramedullary leukemia, including chromosomal abnormalities (such as t(8;21), inv(16)), specific cell-surface markers (CD56, CD2, CD4, CD7), leukemia subtype (M2, M4, M5), and a high presenting leukocyte count (17, 18). Consistent with the reported findings, our study observed that the individuals with clear-cut diagnoses exclusively fell into the M4 or M5 subtypes, with all patients exhibiting chromosomal abnormalities, including case 4 with inv(16). Furthermore, bone marrow flow cytometry analysis of case 1 revealed predominant expression of CD56 and CD4.

In the largest meta-analysis including 30 studies over 10 years, only 3 were children with intracardiac mass, and none of those children survived (19). In our study, three patients died (case 2, 3, and 4), while two children survived (case1 and case5). Whether the cause of death was related to early use of cardiotoxic medication and/or failure to prolong drainage cannot be fully determined, as the data are based on limited case reports.

There is no consensus and standard chemotherapy regimen, and hematologists worldwide have used various regimens depending on the patient’s tolerability and cardiac compromise. Several cases report on cardiac myeloid sarcoma have indicated that regimens incorporating etoposide, mitoxantrone, and cytarabine demonstrate partial efficacy (8, 20). Anthracyclines may not be suitable for patients experiencing cardiac tamponade, particularly during induction chemotherapy. Notably, the two surviving children avoided these factors. Hence, it is imperative to exercise individualized considerations and caution in such cases, and advise avoid cardiotoxic drugs in the early induction chemotherapy period.

While the fractionated radiation up to 24 Gy has demonstrated efficacy in providing symptomatic relief and consolidation of treatment (21), it is not suitable for the majority of children due to the long-term known side effects of radiation. A more moderate approach involving a combination of chemotherapy and molecularly targeted drugs is needed to locally control the disease and prevent bone marrow relapse (22, 23). Recently improvements in AML characterization have led to the incorporation of many novel agents targeting genomic lesions into current treatment strategies or are under investigation (24). Compared to traditional chemotherapy, these new therapies not only reduce the risk of infection but also enhance outcomes in certain subgroups (22, 25). Gilteritinib for the treatment of recurrent AML with FLT3 mutations in children has shown feasibility and efficacy, but the appropriate pediatric dose of the drug is still being explored (26). The established role of HSCT is well-documented in cases of relapsed/refractory and high-risk AML. Currently, the patient is undergoing oral maintenance therapy with Venetoclax and Gilteritinib, and there has been sustained remission of both bone marrow and MS for more than two years. Therefore, a multidisciplinary team approach and precise histopathologic diagnosis, including cytogenetics, are deemed crucial for achieving a successful outcome in the management of rare diseases.

## 5 Conclusions

Early and timely pericardial puncture and drainage, combined with the clinical manifestations and signs, are the key factors in alleviating cardiac tamponade and preventing death. Echocardiographic imaging stands as the primary non-invasive diagnostic tool for detecting cardiac tamponade within the heart, playing a pivotal role in guiding subsequent catheter-based invasive diagnostic approaches. Pericardial puncture and drainage can relieve pericardial tamponade and facilitate cytopathological smear and flow cytometry immunotyping of pericardial effusion to achieve rapid diagnosis. Further research is warranted to identify novel molecular markers for diagnosis and to formulate efficacious therapy protocols.

## Data availability statement

The original contributions presented in the study are included in the article/supplementary material. Further inquiries can be directed to the corresponding authors.

## Ethics statement

The studies involving humans were approved by Shenzhen Children’s Hospital Ethics Committee. The studies were conducted in accordance with the local legislation and institutional requirements. Written informed consent for participation in this study was provided by the participants’ legal guardians/next of kin. Written informed consent was obtained from the individual(s), and minor(s)’ legal guardian/next of kin, for the publication of any potentially identifiable images or data included in this article.

## Author contributions

TL: Investigation, Validation, Writing – original draft, Writing – review & editing. XT: Data curation, Investigation, Methodology, Validation, Writing – original draft. XH: Data curation, Methodology, Visualization, Writing – original draft. LZ: Data curation, Investigation, Resources, Visualization, Writing – original draft. YZ: Resources, Visualization, Writing – original draft. LW: Investigation, Methodology, Validation, Writing – original draft. SLL: Methodology, Validation, Writing – original draft. GZ: Data curation, Investigation, Writing – original draft. FW: Investigation, Methodology, Visualization, Writing – original draft. SXL: Investigation, Validation, Writing – original draft. HM: Funding acquisition, Supervision, Writing – review & editing. YW: Funding acquisition, Supervision, Validation, Writing – review & editing.
